# Research on the formation mechanism of social media burnout among college students based on the ISM-MICMAC model

**DOI:** 10.1038/s41598-026-42958-2

**Published:** 2026-03-08

**Authors:** Jie Wen, Huilin Wang, Huimin Chen

**Affiliations:** 1 Suzhou University of Technology, Suzhou, China; 2 Suzhou Polytechnic Institute of Agriculture, Suzhou, China; 3 Jiangsu Vocational College of Agriculture and Forestry, Jurong, China

**Keywords:** ISM-MICMAC model, Social media burnout, College students, Formation mechanism, Driving factors, Social-psychological factors, Health occupations, Fatigue

## Abstract

This study employs the ISM–MICMAC framework to investigate the structural architecture of social media burnout among university students, aiming to delineate the hierarchical interdependencies and logical pathways of its contributing factors. Utilizing a Delphi-based synthesis, 15 critical variables were identified across individual, psychological, technological, and informational dimensions. The Interpretive Structural Modeling (ISM) results categorize these factors into four distinct levels, unraveling a structural progression where platform-technical mechanisms and deep-seated psychological responses function as the foundational drivers. Complementary MICMAC analysis reveals that factors such as algorithm recommendations and social comparison occupy the Independent Cluster, exerting high driving power over subsequent perceived overloads. This research provides a systematic roadmap for understanding the intricate formation of burnout, shifting the focus from mere symptom management to addressing the root-level structural pressures within digital social environments.

## Introduction

With the rapid development of Internet technology, 5G information technology represented by intelligence and informatization has been integrated into all aspects of life and work. With the development of mobile media technology, the traditional method of social interaction is also undergoing a qualitative change. In real society, the mode of interpersonal communication is also migrating with the change of network communication^1,2^.Social media provides convenient communication channels, rich information resources, and a stage for self-display for college students, becoming an important tool for them to expand social circles, acquire knowledge, and shape self-identity. However, with the deeper use of social media and the increased frequency and duration of social media use, social media burnout is becoming more and more common among college students. Users are also troubled by information overload^3^, service overload^4^, social overload^5^, privacy disclosure^6,7^, etc.

Burnout is a subjective psychological feeling state involving both emotional and cognitive processes^8^.Existing scholarship has examined social media burnout through three primary dimensions: (1) Psychological Factors: Studies emphasize individual traits such as social comparison, self-efficacy, and fear of missing out (FoMO) as significant predictors of emotional exhaustion. (2) Social Environmental Factors: Research highlights the role of social pressure, social norm expectations, and the necessity of maintaining online social capital, which often lead to social overload. (3) Media Usage Behavior and Platform Characteristics: Factors like information overload, algorithm-driven content delivery, and service multiplicity have been identified as external stressors that trigger fatigue.Existing studies have explored individual factors (e.g., social comparison^9^ and technical factors (e.g., algorithm mechanisms^10^ associated with social media burnout, but lack systematic analysis of the hierarchical relationships and interaction pathways among these factors^11^. To address the research gap, this study adopts the ISM-MICMAC model. ISM clarifies hierarchical relationships among factors^12^, while MICMAC analyzes their driving forces and dependencies. This combination enables qualitative and quantitative analysis of the formation mechanism, complementing previous studies’ lack of systematic factor correlation analysis^13^.

## Social media burnout

### Influencing factors of social media burnout among college students

Social media burnout among college students is influenced by a combination of various factors, which are intertwined and collectively impact their social media usage experience. The main factors affecting social media burnout among the college students individual perceptions, individual psychology, platform technology, and information^14^.

### Theoretical applicability of ISM-MICMAC model

While methods like Structural Equation Modeling (SEM) are robust for testing predefined causal hypotheses, they require large sample sizes and established theoretical frameworks. In contrast, the ISM-MICMAC approach is more suitable for this study as it allows for the exploration of a complex system’s internal structure without prior assumptions of causality. It effectively deconstructs the ‘black box’ of burnout formation into a visible hierarchical model, identifying which factors serve as fundamental drivers versus surface-level symptoms.

The combination of the ISM-MICMAC model can comprehensively analyze the formation mechanism of social media burnout behavior of college students from both qualitative and quantitative dimensions. The hierarchical structure analysis of the ISM model provides a basic framework for the quantitative analysis of The Micmac model, and clarifies the relationship and hierarchy of various factors; The driving force dependency matrix construction of The MICMAC model further deepens the understanding of the role of various factors, helps us accurately identify the key factors and secondary factors, and provides a scientific basis for formulating targeted intervention strategies.

## Research methods and data sources

### Data collection and processing

To ensure the rigor of the structural analysis, this study followed a structured three-phase procedure: (1) Factor identification via the Delphi method; (2) Hierarchical structure mapping using the ISM model; and (3) Classification of factors via MICMAC analysis.

When determining the initial influencing factors, we invited eight experts, mainly consisting of two journalism professors, four psychology experts, and two social media operation experts. Mainly based on the following criteria: (1) having more than 5 years of experience in social media research, youth psychology, or platform operations; (2) Publish 3 or more papers in related fields (2018–2024); (3) Familiar with adolescent behavior. Through three rounds of Delphi process, in the first round, a literature review of 58 papers from Web of Science/CNKI was integrated, and 20 initial factors were proposed; In the second round, experts rate the importance of factors on a scale of 1–5 points; Exclude factors with an average value < 3. In the third round, consensus was reached on 15 factors (> 80% consensus) in Table 1.

**Table 1:** Factors influencing social media burnout.

Attribute	Influence Factor	Describe
1. Information Factor^3,7,13–19^	Information Overload(S1)	The state where the volume of received information exceeds an individual’s processing capacity.
	Service Overload(S2)	The exhaustion influenced by managing too many platform-provided services and notifications simultaneously.
2. Individual Perception^15,19–23^	Social Overload(S3)	The perception that social maintenance demands (replying, liking) exceed one’s energy or time.
	Social Trivialization(S4)	The feeling that online social interactions lack depth and have become repetitive or meaningless.
	Self-expression Constraint(S5)	The pressure to self-censor or curate an “ideal self” due to audience collapse or social expectations.
	Privacy Concerns(S6)	The persistent worry about unauthorized access, leakage, or misuse of personal data on platforms.
3. Platform Functions^10,25^	Feedback Mechanism(S7)	The interactive design (likes, shares, comments) that drives constant user engagement and validation-seeking.
	Algorithm Recommendation(S8)	The personalized content delivery logic that keeps users hooked through continuous data-driven stimuli.
	Service Multiplicity(S9)	The integration of diverse functions (shopping, news, gaming) within one app, increasing cognitive burden.
4. Individual Psychology^8,13,16,24–26^	Social Comparison(S10)	The tendency to evaluate one’s self-worth by comparing one’s life with the idealized lives of others online.
	Group Identity(S11)	The psychological pressure to conform to group norms and maintain a sense of belonging within online circles.
	Self-efficacy(S12)	One’s belief in their ability to manage social media use and resist impulsive digital consumption.
	Fear of Missing Out (FoMO)(S13)	The pervasive anxiety that others might be having rewarding experiences from which one is absent.
	Social Norm Pressure(S14)	The perceived pressure from society or peers to stay “always-on” and immediately responsive.
	Withdrawal and Avoidance(S15)	The behavioral outcome of burnout characterized by reducing usage, deactivating accounts, or ghosting.

### Construction steps of the ISM-MICMAC model

On the basis of the data collection and processing, we constructed an ISM-MICMAC model according to these steps to analyze the formation mechanism of the social media burnout behavior of college students.

#### Building the adjacency matrix

The 15 extracted factors affecting social media volumes are encoded and named, as shown in Table 1. On this basis, the adjacency matrix A is determined according to the construction rules of the adjacency matrix. To avoid subjective errors in the final results, experts are invited to score the correlation degree between the identified hidden barriers, and the rules are constructed as follows: if more than half of the experts believe that F_i_ has a direct impact on F_j_, the result is taken as 1, otherwise it is taken as 0. Combined with the results of the expert interviews and discussions and the subsequent comprehensive analysis, the adjacency matrix of the influencing factors of social media burnout is obtained, as shown in Table 2.

**Table 2:** The Adjacency matrix A.

Factor	S1	S2	S3	S4	S5	S6	S7	S8	S9	S10	S11	S12	S13	S14	S15
S1	0	1	0	0	0	0	0	0	0	0	0	0	0	0	0
S2	0	0	0	0	0	0	0	0	0	0	0	0	0	0	1
S3	1	0	0	1	0	0	0	0	0	0	0	0	0	0	0
S4	0	1	0	0	0	0	0	0	0	0	0	0	0	0	1
S5	0	0	1	0	0	1	0	0	0	0	0	0	0	0	0
S6	0	0	0	0	0	0	0	0	0	0	0	0	0	0	1
S7	0	0	0	0	0	0	0	0	1	1	0	0	0	0	0
S8	0	0	0	0	0	0	1	0	1	0	1	0	1	0	0
S9	1	1	0	0	0	0	0	0	0	0	0	1	0	0	0
S10	0	0	1	1	0	0	0	0	0	0	1	0	0	0	0
S11	0	0	0	0	1	0	0	0	0	0	0	1	0	0	0
S12	0	0	0	0	0	0	0	0	0	0	0	0	1	1	0
S13	1	0	0	0	0	0	0	0	0	1	0	0	0	1	0
S14	0	0	0	0	0	1	0	0	0	0	0	0	0	0	1
S15	0	0	0	0	0	0	0	0	0	0	0	0	0	0	0

#### Calculation of the reachable matrix

Through the adjacency matrix, we can intuitively observe the direct influence relationship between different hidden obstacles. However, the indirect relationship between them cannot be reflected. Therefore, MATLAB software is used to calculate the reachable matrix by performing Boolean algebra operations on the adjacency matrix A. When a matrix M satisfies formula (1), it is the reachable matrix, as shown in Table 3.

1 $$\mathrm{M}={\mathrm{(A+}\mathrm{I}\mathrm{)}}^{\mathrm{n+1}}={\mathrm{(A+}\mathrm{I}\mathrm{)}}^{\mathrm{n}}\neq{\mathrm{(A+}\mathrm{I}\mathrm{)}}^{\mathrm{n-1}}$$

**Table 3:** The Reachable matrix M.

Factor	S1	S2	S3	S4	S5	S6	S7	S8	S9	S10	S11	S12	S13	S14	S15	D_i_
S1	1	1	0	0	0	0	0	0	0	0	0	0	0	0	0	2
S2	0	1	0	1	0	0	0	0	0	0	0	0	0	0	0	2
S3	1	1	1	0	0	0	0	0	0	0	0	0	0	0	0	3
S4	0	1	0	1	0	1	0	0	0	0	0	0	0	0	0	3
S5	0	0	0	1	1	0	0	0	0	0	0	0	0	0	0	2
S6	0	0	1	0	1	1	0	0	0	0	0	0	0	1	0	4
S7	1	1	1	1	0	0	1	0	1	1	1	0	0	0	0	8
S8	1	1	1	1	1	0	1	1	1	1	0	0	0	0	0	9
S1	1	1	1	0	0	1	0	0	1	0	1	1	0	0	0	7
S10	1	0	0	1	1	0	0	0	0	1	1	0	1	0	0	6
S11	0	1	1	1	0	0	0	0	0	0	1	1	0	0	0	5
S12	0	1	0	0	1	0	0	0	0	0	0	1	1	1	0	5
S13	1	0	0	1	1	0	0	0	0	1	0	1	1	0	0	6
S14	0	0	0	0	1	1	0	0	0	1	0	1	0	1	1	6
S15	0	0	0	0	0	0	0	0	0	0	0	0	0	0	1	1
R_j_	9	9	7	8	7	4	3	2	3	5	5	5	4	4	2	/

#### Decomposition of reachable matrix M

Based on the reachability matrix, each factor is categorized into different levels. The reachable set R(Si) of influencing factor Si refers to the set of influencing factors corresponding to the columns in the reachability matrix where all elements in the Si-th row are 1. The antecedent set Q(Si) of influencing factor Si refers to the set of influencing factors corresponding to the rows in the reachability matrix where all elements in the Si-th column are 1. When R(Si)∩Q(S) = R(Si), the factor can be selected hierarchically. After extraction, the rows and columns corresponding to the extracted factor are temporarily removed from the reachability matrix. This process is repeated iteratively until all factors in the reachability matrix have been extracted.

**Fig. 1:**
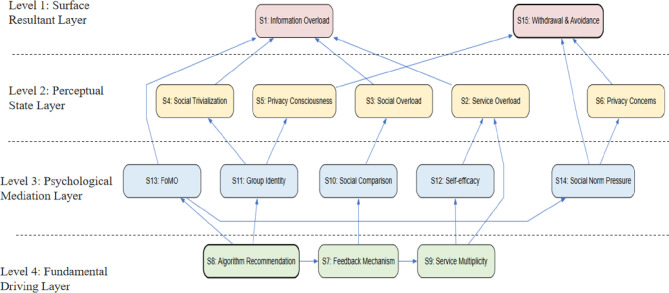
Hierarchical structure and association relationship analysis diagram of elements.

Finally, Through continuous screening, we divided the 15 influencing factors into four levelsas shown in Fig. 1. The factors at the highest level are usually the final result of social media burnout, while the underlying factors are some root influencing factors. We remove the redundant relationships with transitivity in the reachable matrix to obtain a more concise skeleton matrix, which more intuitively displays the essential connections between various factors.

## Result analysis

### Hierarchical structure analysis of influencing factors

Based on the reachability matrix and the level-partitioning process, a four-level Interpretive Structural Model (ISM) was generated (see Fig. 1). This hierarchy illustrates the multi-stage transmission path from foundational technical/social triggers to terminal burnout behaviors.

#### Surface-level direct factors (Level 1)

At the highest level (Level 1), Information Overload (S1) and Withdrawal and Avoidance (S15) are identified as the most immediate manifestations of social media burnout. In Fig. 1, these factors represent the terminal symptoms where the cumulative pressures from lower levels finally erupt into behavioral consequences. S1 serves as the cognitive threshold, while S15 acts as the behavioral response. these factors exhibit high dependence, meaning they are the “end-of-pipe” results of the entire causal chain.

#### Indirect intermediate factors (Level 2)

The second level (Level 2) consists of Service Overload (S2), Social Overload (S3), Social Trivialization (S4), Functional Complexity (S5), and Privacy Concerns (S6). These factors serve as the “perceptual bridge” between psychological strain and terminal burnout. Figure 1 shows that when students are exposed to complex platform functions (S5) and excessive social demands (S3), their perception of environmental stress intensifies, directly feeding into the Level 1 symptoms. This layer reflects the structural burden of the digital environment, acting as the primary channel through which deep-seated stressors are converted into immediate cognitive fatigue.

#### Psychological strain and social drivers (Level 3)

The third level (Level 3) acts as the “internalization layer,” comprising Social Comparison (S10), Group Identity (S11), Self-efficacy (S12), Fear of Missing Out (S13), and Social Norm Pressure (S14). This layer highlights the social-psychological dimension of burnout. As depicted in Fig. 1, the anxiety of being disconnected (S13) and the pressure of social norms (S14) create a constant state of mental strain. These factors are not only influenced by the root drivers at the bottom but also dictate the intensity of the overload perceived at Level 2. The placement of these factors here explains why social media burnout is more than just a technical issue—it is deeply rooted in the social interaction needs of university students.

#### Deep-seated root factors (Level 4)

At the base of the model (Level 4), Feedback Mechanisms (S7), Algorithm Recommendation (S8), and Group Pressure (S9) are the fundamental root factors. These are the “prime movers” with the strongest driving power in the entire system. Figure 1 reveals that the platform’s algorithmic logic (S8) and reward-based feedback loops (S7) are the exogenous sources that trigger the entire hierarchy. These factors are characterized by high driving power and low dependence, meaning they are the most stable and decisive elements. Consequently, any long-term mitigation of social media burnout must prioritize the ethical redesign of these fundamental technical and social architectures.

### Classification and driving mechanism of factors based on MICMAC analysis

**Fig. 2:**
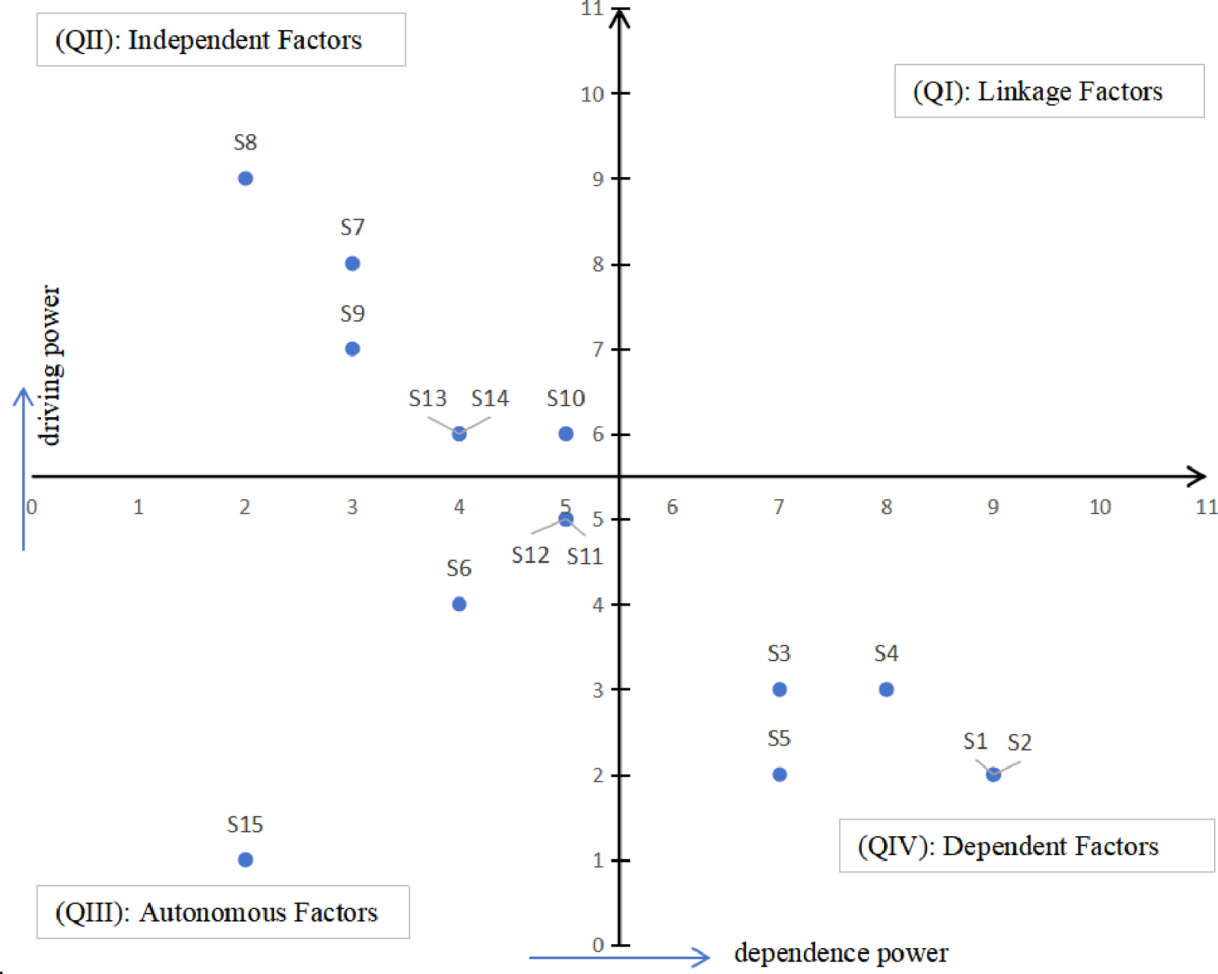
Classification of the driving force dependence of influencing factors.

The MICMAC method is employed to identify the key factors by analyzing their driving power and dependence power. The driving power indicates the degree of influence a factor exerts over others, calculated as the horizontal sum of the elements in the reachability matrix. The dependence power represents the extent to which a factor is influenced by others, derived from the vertical sum of the elements in the matrix.In this study, the 15 identified factors are mapped into a four-quadrant classification based on their calculated coordinates (D, R). The distribution of these factors is illustrated in Fig. 2.

#### Linkage cluster (Quadrant I: High DP, High DEP)

Factors in Quadrant I are characterized by both high driving power and high dependence, representing the most unstable and sensitive elements of the system. Interestingly, in our empirical analysis, Quadrant I remains vacant. The absence of factors in the Linkage Cluster suggests that the system lacks “bridge” variables that are simultaneously highly influenced and highly influential. This implies that the transition from root influences to burnout behaviors does not rely on a single set of unstable linkage factors, but rather follows a more direct causal path.

#### Independent cluster (Quadrant II: High DP, Low DEP)

Quadrant II includes factors with high driving power but low dependence, identified here as Feedback Mechanisms (S7), Algorithm Recommendation (S8), Group Pressure (S9), Social Comparison (S10), FoMO (S13), and Social Norm Pressure (S14). These factors act as the “engine room” of the burnout mechanism. Because they possess strong driving energy while remaining relatively unaffected by other variables, they serve as primary initiators of the burnout process. For instance, Algorithm Recommendation (S8) and Social Comparison (S10) function as core drivers: algorithms push content that triggers comparison, which intensifies psychological strain without being easily mitigated by other system factors. These independent factors are the key entry points for intervention.

#### Autonomous cluster (Quadrant III: Low DP, Low DEP)

Factors in this quadrant exhibit low driving power and low dependence, signifying a degree of isolation from the core causal chain. Our results place Privacy Concerns (S6), Group Identity (S11), Self-efficacy (S12), and Withdrawal/Avoidance (S15) in this cluster. While S15 is the terminal behavior of burnout, its placement here indicates that once a student reaches the stage of “Withdrawal,” the behavior itself has limited power to trigger further new stressors within this specific model. These factors represent “stabilized states” or individual-level perceptions that, while relevant to the personal experience, do not aggressively drive the system’s evolution or respond strongly to other systemic shifts.

#### Dependent cluster (Quadrant IV: Low DP, High DEP)

The Dependent Cluster consists of factors with low driving power but high dependence, representing the “outputs” or “symptoms” of the system. This cluster includes Information Overload (S1), Service Overload (S2), Social Overload (S3), Social Trivialization (S4), and Functional Complexity (S5). These factors are the “recipients” of the systemic pressure. Although they are the most visible manifestations of the burnout experience (the “overload” sensations), they are strongly driven by the factors in Quadrant II. Their high dependence suggests that to alleviate these overloads, interventions must target the root drivers in the Independent Cluster rather than treating these symptoms in isolation.

The analysis reveals that the ‘Five Overloads’ (S1–S5) function as the terminal manifestations of the burnout process rather than its foundational triggers. Their positioning in Quadrant IV (High DEP and Low DP) confirms that these perceived overloads are endogenous symptoms highly dependent on the broader systemic dynamics—they are the cumulative results of structural pressures rather than independent causes. In this mechanism, the Independent drivers in Quadrant II (such as Algorithm Recommendation and Social Comparison) exert continuous upward pressure, acting as the primary impetus that eventually manifests as the debilitating overloads observed in the Dependent cluster. Thus, the ‘Five Overloads’ represent the point where systemic stressors are internalized by the user, marking the transition from external platform dynamics to individual psychological exhaustion.

## Conclusions and countermeasures

This study develops a hierarchical structural framework to explore the formation mechanism of social media burnout among college students via the ISM-MICMAC method. The results systematically delineate the logical architecture of inter-factor dependencies:

### Social media burnout as a multi-level structural progression

The model identifies Independent Drivers (S7–S10, S13–S14)—encompassing both platform-technical functions and deep-seated psychological mechanisms—as the foundational layer of the hierarchy. These factors are perceived to sustain the initial impetus that leads to Dependent Overloads (S1–S5). Finally, these overloads culminate in the top-level outcome of Withdrawal and Avoidance (S15), forming a logical pathway from systemic drivers to behavioral symptoms.

### Strategic factors like algorithm recommendation (S8) and feedback mechanisms (S7) exhibit the highest driving power

Positioned in the Independent Cluster (Quadrant II), they function as the “systemic engine.” While these factors enhance user engagement, they simultaneously generate the structural strain that permeates the entire hierarchy. Their high driving power suggests that they are the primary logical precursors to the subsequent psychological and perceptive states.

### Withdrawal and avoidance (S15) serves as the terminal stabilized state

In the structural model, S15 represents the final manifestation of burnout. This positioning indicates that burnout is more than a fleeting negative emotion; it is a cumulative result of multi-dimensional pressures, acting as a psychological self-preservation mechanism adopted by college students to prevent further depletion of cognitive resources.

It is essential to clarify that the structural relationships rooted in the systematic consensus of domain experts. While the ISM–MICMAC approach provides a rigorous qualitative mapping of the inter-factor influence logic, it captures perceived causality rather than empirically tested statistical effects. Therefore, while these findings offer a robust framework for understanding the systemic drivers of social media burnout, future research should employ quantitative methods, such as Structural Equation Modeling (SEM), to verify the magnitude and significance of these paths using large-scale empirical data.

## Data Availability

The datasets used and/or analysed during the current study available from the corresponding author on reasonable request.
